# Frameless fractionated stereotactic radiation therapy of intracranial lesions: impact of cone beam CT based setup correction on dose distribution

**DOI:** 10.1186/1748-717X-8-153

**Published:** 2013-06-25

**Authors:** Petra Maria Haertl, Rainer Loeschel, Natalia Repp, Fabian Pohl, Oliver Koelbl, Barbara Dobler

**Affiliations:** 1 Department of Radiotherapy, Regensburg University Medical Center, Regensburg, Germany; 2 Department of Computer Science and Mathematics, University of Applied Sciences, Regensburg, Germany

**Keywords:** CBCT, Setup error, Reproducibility, Daily dose distribution, Cumulative dose distribution, Actual dose distribution, Frameless stereotactic radiation therapy, Intracranial lesions

## Abstract

**Background:**

The purpose of this study was to evaluate the impact of Cone Beam CT (CBCT) based setup correction on total dose distributions in fractionated frameless stereotactic radiation therapy of intracranial lesions.

**Methods:**

Ten patients with intracranial lesions treated with 30 Gy in 6 fractions were included in this study. Treatment planning was performed with Oncentra® for a SynergyS® (Elekta Ltd, Crawley, UK) linear accelerator with XVI® Cone Beam CT, and HexaPOD™ couch top. Patients were immobilized by thermoplastic masks (BrainLab, Reuther). After initial patient setup with respect to lasers, a CBCT study was acquired and registered to the planning CT (PL-CT) study. Patient positioning was corrected according to the correction values (translational, rotational) calculated by the XVI® system. Afterwards a second CBCT study was acquired and registered to the PL-CT to confirm the accuracy of the corrections. An in-house developed software was used for rigid transformation of the PL-CT to the CBCT geometry, and dose calculations for each fraction were performed on the transformed CT. The total dose distribution was achieved by back-transformation and summation of the dose distributions of each fraction. Dose distributions based on PL-CT, CBCT (laser set-up), and final CBCT were compared to assess the influence of setup inaccuracies.

**Results:**

The mean displacement vector, calculated over all treatments, was reduced from (4.3 ± 1.3) mm for laser based setup to (0.5 ± 0.2) mm if CBCT corrections were applied. The mean rotational errors around the medial-lateral, superior-inferior, anterior-posterior axis were reduced from (−0.1 ± 1.4)°, (0.1 ± 1.2)° and (−0.2 ± 1.0)°, to (0.04 ± 0.4)°, (0.01 ± 0.4)° and (0.02 ± 0.3)°. As a consequence the mean deviation between planned and delivered dose in the planning target volume (PTV) could be reduced from 12.3% to 0.4% for D_95_ and from 5.9% to 0.1% for D_av_. Maximum deviation was reduced from 31.8% to 0.8% for D_95_, and from 20.4% to 0.1% for D_av_.

**Conclusion:**

Real dose distributions differ substantially from planned dose distributions, if setup is performed according to lasers only. Thermoplasic masks combined with a daily CBCT enabled a sufficient accuracy in dose distribution.

## Background

In stereotactic radiation therapy a steep dose gradient is required to spare healthy tissue. As a consequence patient setup has to be highly precise and reproducible. During the last years, on board Cone Beam CT (CBCT) systems became commercially available for accurate patient positioning and made frameless stereotactic radiation therapy (SRT) possible. The advantage of SRT as compared to frame based systems is the fast setup procedure with better patient comfort. Previous studies investigated the magnitude of setup errors depending on different positioning systems [1-4]. Boda-Heggeman et al. [1] analysed differences in accuracy between rigid and thermoplastic (TP) mask systems. Rotational and translational displacements were investigated. TP masks, in combination with CBCT, reached similar accuracy as rigid mask systems. Gilbeau L et al. [2] compared the setup accuracy of different TP masks. The investigation was performed by use of portal imaging. No substantial difference between the masks was found. Tryggestad et al. [4] evaluated setup accuracy of four frameless TP mask systems using daily CBCT. All mask systems examined within this study were regarded as suitable for SRT, if daily CBCT was performed. A quality assessment of SRT by means of two different CBCT was performed by Peng et al. [3]. The impact of setup errors on the daily and total dose distribution in the patient was, however, not subject of these studies.

The influence of rotational setup errors to generalized equivalent uniform dose (gEUD) was evaluated by Beltran et al. [5] for three cases of pediatric brain tumours. Rotational errors were considered by alteration and recalculation of the initial treatment plan (TPL), to simulate rotations of 2° and 4° around each cardinal direction separately. Rotation of 2° changed gEUD values of planning target volumes (PTV) and organs at risk (OAR) less than 2%. Rotations of 4° led to the same result to parallel type normal structures, whereas in case of serial type normal structures and PTV the gEUD values changed by 5% and 10%. Consequently a correction of rotational errors of more than 2° was advised.

Guckenberger et al. [6] found only a small influence of setup error to dose, if the tumour was located in base of skull. Deviation in dose was less than 5%. A recent report investigated dosimetric influence of translational and rotational setup errors to dose by single fraction SRT [7]. Result of this study is a need of accuracy of 1 mm or less to avoid a decreased target coverage and a loss of dose conformity > 5% with respect to TPL.

In case of fractionated SRT daily uncertainties add up and may cause deviations in the dose distribution compared to TPL. In contrary to single fraction SRT, random errors in setup may lead to a more shallow dose gradient, whereas systematic errors cause a shift and rotation of the dose distribution. The aim of this study was to investigate the influence of laser and CBCT based setup technique on the total dose in fractionated SRT.

## Methods and material

### Patients and dose prescription

Data of 10 intracranial lesions were retrospectively evaluated in this study. Dose prescription was individually adapted to clinic (Table 1).

**Table 1:** Dose prescription and normalization for each localization

**Localization**	**Normalized**	**Percent**
1	Average of PTV	100
2	Average of PTV	100
3	Maximum of PTV	90
4	Average of PTV	100
5	Maximum of PTV	90
6	Maximum of PTV	90
7	Isocenter	100
8	Average of PTV	100
9	Maximum of GTV	90
10	Maximum of GTV	90

### Treatment unit and treatment planning system (TPS)

The treatment unit consists of a SynergyS® (Elekta Ltd, Crawley, UK) linear accelerator with a BeamModulator™ head, leaf width of 4 mm at isocenter. For image guided radiation therapy (IGRT), a XVI® Cone Beam CT (CBCT) and a HexaPOD™ couch top in connection with software iGuide® Ver.R1.1.Rev1 (Medical Intelligence, Schwabmünchen, Germany) were used. Dose calculations were performed in Oncentra® External Beam v4.0 SP1 (Nucletron BV, Veenendaal, Netherlands) (OMP) with a pencil beam algorithm. Beam quality was 6MV.

### Immobilization and setup protocol

Patients were immobilized using a stereotactic TP mask system (BrainLab) or TP mask system (Reuther). After initial patient setup with respect to lasers, a CBCT study was acquired and registered to the planning CT study. Translational deviations in medial-lateral, superior-inferior, anterior-posterior direction (Δx, Δy, Δz) and rotational deviations around x, y, z (Δα, Δβ, Δγ) were calculated by the XVI® software. Rotational and translational displacement was corrected. A second CBCT study was acquired and the actual deviation was determined.

### Evaluation of setup errors

Setup deviation after laser setup and CBCT was recorded for each fraction in 6 degrees of freedom. The absolute values of the translational displacement vectors were calculated. The systematic and random setup errors were calculated as the mean setup error and its standard deviation (SD) [8] for each patient over all fractions for a) setup to lasers only and b) CBCT corrected patient setup. The group systematic error, the mean of all means and the group mean of the SD (random error) calculated as the root mean square of the SD’s of all patients [8] were determined as well.

### Transformation of DICOM data

For assessment of the dose distribution achieved when residual translational and rotational setup errors are present after patient setup, the planning CT data were transformed according to the setup errors calculated by the XVI system for all therapy fractions. For each fraction, the original TPL was then recalculated on the transformed CT data set, resulting in a dose distribution of the fraction. The total dose distribution is then achieved by inverse transformation and summation of the dose distributions of each fraction.

DICOM data can in principle be transformed by a transformation of the reference coordinate system. Since Oncentra®, however, does not allow the import of CT data with a rotated reference coordinate system, the DICOM data had to be transformed keeping the original reference coordinate system. This was achieved by target to source mapping to avoid voids in the target image [9]. An empty CT cube is created and for each voxel the corresponding coordinates in the source image are determined by inverse transformation of the voxel coordinate. The grey value is calculated from the neighbouring voxels of the source coordinate by trilinear interpolation, the expansion of bilinear interpolation to three dimensions [9,10]. The dose distribution for each fraction is then calculated on the corresponding transformed CT cube and stored in a dose cube. To be able to calculate the cumulative dose distribution for all fractions, the dose cubes of each fraction are transformed back to the original geometry such, that all fractional dose cubes are of the same size and resolution. The dose cubes are then summed up by summation of the corresponding dose values of each individual voxel to achieve the cumulative dose value over all fractions. The cumulative dose cube can then be imported to the original planning CT in Oncentra® for comparision with the planned dose distribution.

The geometrical transformation has been validated on a geometrical cubical phantom with asymetric rods inserts, which was originally designed for quality assurance of patient positioning by CBCT. Registration of the transformed CT data with the planning CT in XVI confirmed the values used for translation and rotation of the data cube. The influence of the interpolation on the final dose distribution depends on the slope in grey values of the CT data used. It was tested on sample patient CT data as follows: In Oncentra a transformation of the CT data was simulated by a translation of the isocenter of the treatment plan of −1.0 cm in each direction and a rotation of the couch and the gantry around −4.0° to simulate a rotation around the cranio-caudal and the ventro-dorsal axis. A rotation around the third axis cannot be simulated in Oncentra and is therefore not taken into account. The corresponding translation of 1.0 cm and 4.0° rotation of the CT data was performed using the in-house software. The resulting CT data set was imported in Oncentra for recalulation of the plan. The dose distributions achieved with the two methods were compared in the evaluation software OmniPro-I’mRT (IBA Dosimetry, Schwarzenbruck, Germany) for the CT slices in the high dose region. For all slices absolute values of the dose differences were within 0.4% ± 1.1%.

### Evaluation of impact to dose

The total dose distributions which would be achieved with setup to light markers only and which are achieved with CBCT based set up were then compared to the original dose distribution of the TPL. For the PTV D_95_ (dose to 95% of PTV), D_05_ (dose to 5% of PTV), and D_av_ (average dose to PTV) were evaluated. To quantify changes in dose distribution in spite of different normalization, relative deviations to the corresponding TPL were calculated and compared. For assessment of how well the prescription dose conforms to the size and the shape of the target, the Paddick conformity index CI_PTV_[11] was calculated by CI_PTV_ = (PTV_PD_/PTV) * (PTV_PD_/Vol_PD_), with the volume of PTV treated with the prescription dose PTV_PD_, and the total volume receiving the prescription dose Vol_PD_. The ideal, i.e. most conformal TPL has a CI_PTV_ of 1. The lower the value of CI_PTV_, the larger is the mismatch between Vol_PD_ and PTV, i.e. the less conformal the plan.

A comparison of the dose to certain organs at risk (OAR) over all tumour localizations was not considered meaningful because of the different distances of PTV to OAR and the low absolute doses achieved in the TPLs.

## Results

### Setup error

The use of CBCT decreased the displacement vector (overall mean ± SD) from (4.3 ± 1.3) mm to (0.5 ± 0.2) mm as compared to laser setup.

**Figure 1:**
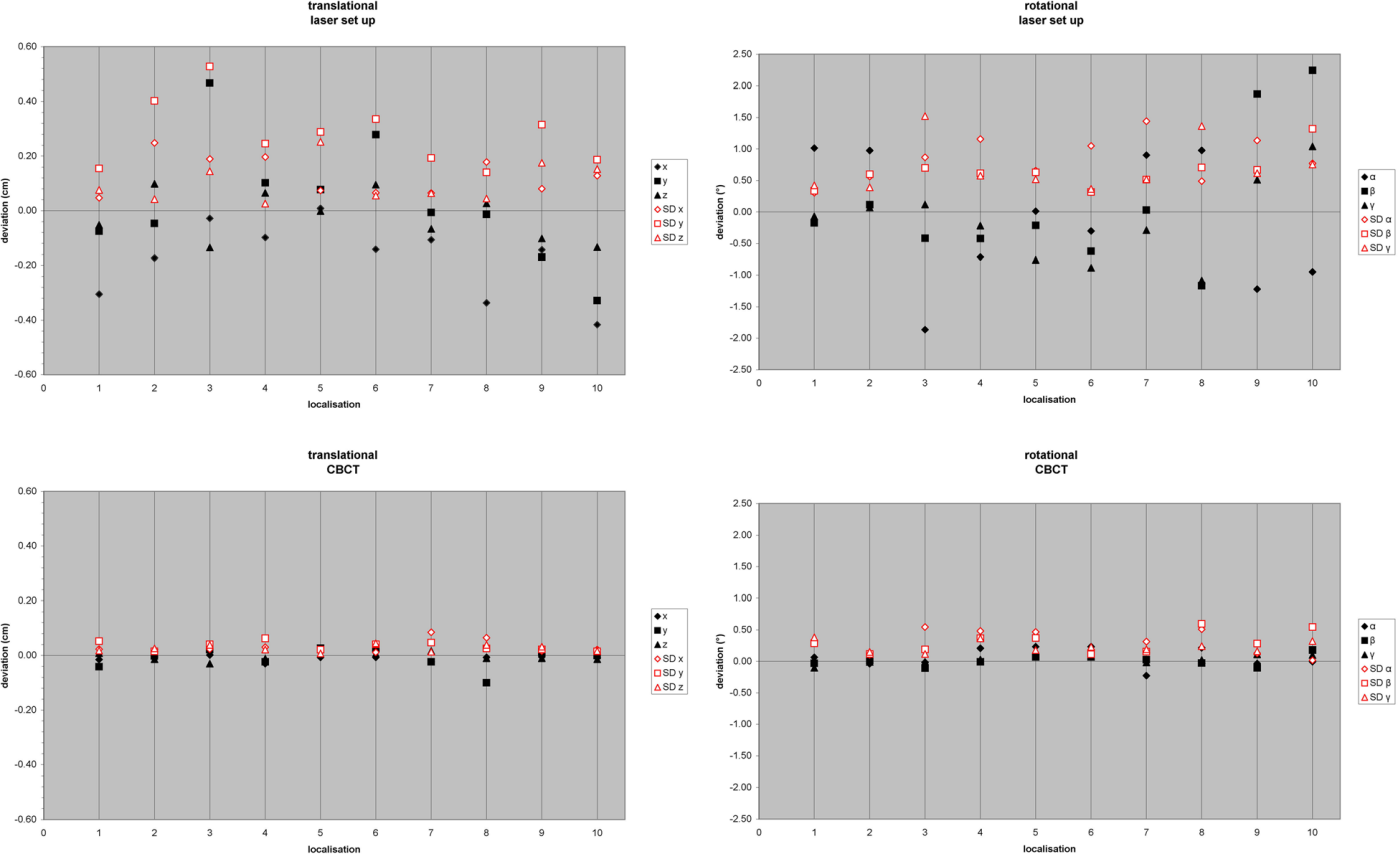
Average translational (left) and rotational (right) setup error and SD for each lesion for laser based (top) and CBCT based (bottom) patient setup.

Average setup error and SD for each lesion in case of laser setup and CBCT is demonstrated in Figure 1. After laser setup a maximum translational systematic displacement of 4.7 mm was measured in y direction (localization 3). The random error in this case and direction was 5.3 mm. The maximum rotational systematic error was 2.2° for β (localization 10), with a random error of 1.3°. The systematic and random error was decreased after CBCT below 1 mm and 0.6° (Figure 1).

The group systematic error, group mean of SD and maximum displacement over all localizations are presented in Table 2.

**Table 2:** Group systematic error (overall mean), group mean SD and maximum displacement

	**Laser setup**			**CBCT setup**		
	**Group mean**	**Group SD**	**Max**	**Group mean**	**Group SD**	**Max**
x (mm)	−1.7	1.4	5.5	−0.1	0.4	2.0
y (mm)	0.3	3.0	9.5	−0.2	0.4	1.6
z (mm)	−0.2	1.2	4.4	0.0	0.3	1.1
α (degree)	−0.1	0.9	−3.1	0.0	0.4	1.3
β (degree)	0.1	0.7	4.2	0.0	0.3	1.2
γ (degree)	−0.2	0.8	−3.7	0.0	0.3	0.8

### Impact on dose distribution

**Figure 2:**
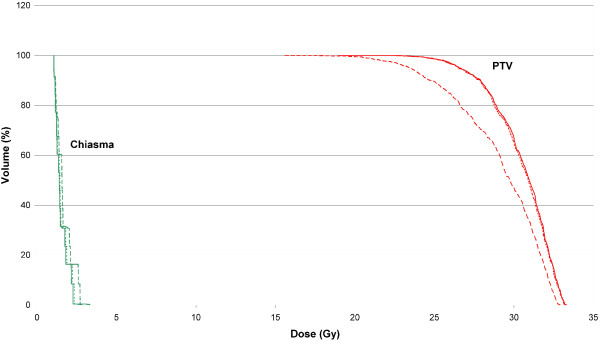
DVH comparison for a typical case: The continuous line represents the original TPL, the dashed line patient setup according to lasers, and the dotted line setup based on CBCT.

Setup errors for a) patient setup according to lasers only and b) CBCT based setup correction lead to differences in dose distribution as compared to the original TPL. The average Paddick conformity index CI_PTV_ changed from 0.67 (min 0.48/max 1.02) in the TPL to 0.39 (min 0.00/max 0.99) for laser based set up, and to 0.65 (min 0.44/max 0.94) for CBCT based setup. Results for PTV parameters D_95_, D_05_ and D_av_ are listed in Table 3. For setup to light markers only, D_95_ and D_av_ decrease substantially, with a maximum deviation of −31.8% (D_95_), -20.4% (D_av_), and −17.7% (D_05_). For CBCT based setup correction only small deviations up to −0.8% (D_95_), -0.4% (Dav), and −3.9% (D_05_) can be observed. Most sensitive to displacements resulting from setup to lasers was D_95_ with a mean deviation of −12.3% followed by D_av_ with a mean deviation of −5.9%. If setup was corrected based on CBCT data, these values were clearly reduced to a mean deviation of −0.4% in D_95_ and −0.1% in D_av_.The influence of displacement on dose to OAR depends on the distance between OAR and PTV and on the dose gradient. Generally all included TPLs contribute only a small amount of dose to OAR. Because the initial doses to OAR were low, absolute dose deviations were negligible. As an example, the dose volume histogram (PL-CT, Laser and CBCT) for localization 9 is presented in Figure 2.

**Table 3:** **Impact on setup technique on D**_**95**_**, D**_**05 **_**and D**_**av **_**of the PTV as compared to the original treatment plan**

**PTV**	**Laser setup**			**CBCT setup**		
	**D95**	**D05**	**Dav**	**D95**	**D05**	**Dav**
mean	−12.3%	−3.9%	−5.9%	−0.4%	−0.5%	−0.1%
max	−31.8%	−17.7%	−20.4%	−0.8%	−3.9%	−0.4%
min	−1.7%	−0.2%	−0.5%	0.2%	0.2%	0.4%

Changes are given in % of the respective dose value of the treatment plan, negative values represent a dose reduction and positive values an increase.

## Discussion

One base of successful stereotactic radiation therapy of intracranial lesions is a reliable patient setup. Intrafractional and interfractional changes in patient setup have to be considered. Intrafractional changes arise from patient motion during treatment. Initially invasive stereotactic frames for rigid immobilisation together with localizers were used for single fraction stereotactic radiation therapy [12]. Stereotactic TP mask systems including localizers were used for fractionated radiation therapy [4]. Both methods were time consuming and uncomfortable for patients. Guckenberger et al. proposed the use of the bony structures of skull instead of localizer, to determine the target position. Comparison of bony registration (CBCT) and soft-tissue match using a mobile CT showed a highly significant correlation for brain metastases [13]. TP masks were evaluated with respect to positioning uncertainties. Several investigations were performed to determine interfractional and intrafractional uncertainties using TP masks combined with CBCT [1,4,13-15]. Mean displacement values and corresponding SD for TP masks in case of laser setup evaluated in our study agreed with the data presented in [1] (4.7 mm ± 1.7 mm) and [13] (4.6 mm ± 2.1 mm).

All these studies showed that setup deviations can be reduced but not completely avoided by the use of immobilization devices in combination with CBCT correction. It is therefore necessary to assess the impact of these residual setup deviations on dose distribution. For single fraction SRT, this has been performed by Guckenberger et al. [7], who determined a loss of dose conformity measured by CI_PTV_ with 0.73 for TPL to 0.43 for laser set up and to 0.73 for CBCT based set up. In this case the impact of residual setup errors is negligible for CBCT based setup but not for laser based setup.

The scope of our study was to evaluate the influence of setup errors on the dose distribution in fractionated stereotactic treatments. In contrary to single fraction SRT, where all kinds of setup errors lead basically to a shift and rotation of the dose distribution, this is only the case for systematic errors in fractionated SRT. Random errors cause a broadening in dose fall off, because dose distributions of all fractions are combined to the total dose. It could be shown, that patient setup based on light markers only would lead to a substantial loss of dose coverage in the PTV even in fractionated SRT, whereas patient setup using CBCT resulted in minor deviations as compared to the TPL, with a CI_PTV_ of 0.67 for the TPL, 0.36 for laser set up, and 0.65 for CBCT based setup. These results confirm the importance of CBCT even in case of fractionated frameless SRT.

## Conclusion

Real total dose distributions in fractionated SRT differ substantially from planned dose distributions, if setup is performed according to lasers only. Frameless fractionated SRT using thermoplasic masks combined with a daily CBCT enables an agreement between planned and delivered dose to PTV within 1% for D_95_ and D_av_.

## Abbreviations

CBCT: Cone Beam CT; FSRT: Frame Based Stereotactic Treatment; gEUD: Generalized Equivalent Uniform Dose; OAR: Organ at Risk; PL-CT: Planning CT; PTV: Planning Target Volume; SD: Standard Deviation; SRT: Frameless Stereotactic Treatment; TP: Thermoplastic Mask; TPL: Treatment Plan; TPS: Treatment Planning System.

## Competing interests

This work was supported by Elekta GmbH, Hamburg, Germany.

## Authors’ contributions

PH carried out the study and drafted the manuscript. RL and NR developed the in-house software for transformation of CT data and dose distribution. FP designed the workflow for CBCT based patient set up. OK helped to draft the manuscript and designed the medical aspects of it. BD participated in the design of the study and coordination of it and helped to draft the manuscript. All authors read and approved the final manuscript.
